# Membrane topologies of the PGLa antimicrobial peptide and a transmembrane anchor sequence by Dynamic Nuclear Polarization/solid-state NMR spectroscopy

**DOI:** 10.1038/srep20895

**Published:** 2016-02-15

**Authors:** Evgeniy Sergeevich Salnikov, Christopher Aisenbrey, Fabien Aussenac, Olivier Ouari, Hiba Sarrouj, Christian Reiter, Paul Tordo, Frank Engelke, Burkhard Bechinger

**Affiliations:** 1 University of Strasbourg/CNRS, UMR7177, Chemistry Institute, 67070 Strasbourg, France; 2 Bruker BioSpin, 34, rue de l’Industrie, 67166 Wissembourg, France; 3 Aix-Marseille University, Institut de Chimie Radicalaire, UMR 7273, Faculté des Sciences, 13397 Marseille, Cédex 20, France; 4 Bruker BioSpin, Silberstreifen, 76287 Rheinstetten, Germany

## Abstract

Dynamic Nuclear Polarization (DNP) has been introduced to overcome the sensitivity limitations of nuclear magnetic resonance (NMR) spectroscopy also of supported lipid bilayers. When investigated by solid-state NMR techniques the approach typically involves doping the samples with biradicals and their investigation at cryo-temperatures. Here we investigated the effects of temperature and membrane hydration on the topology of amphipathic and hydrophobic membrane polypeptides. Although the antimicrobial PGLa peptide in dimyristoyl phospholipids is particularly sensitive to topological alterations, the DNP conditions represent well its membrane alignment also found in bacterial lipids at ambient temperature. With a novel membrane-anchored biradical and purpose-built hardware a 17-fold enhancement in NMR signal intensity is obtained by DNP which is one of the best obtained for a truly static matrix-free system. Furthermore, a membrane anchor sequence encompassing 19 hydrophobic amino acid residues was investigated. Although at cryotemperatures the transmembrane domain adjusts it membrane tilt angle by about 10 degrees, the temperature dependence of two-dimensional separated field spectra show that freezing the motions can have beneficial effects for the structural analysis of this sequence.

Solid-state NMR spectroscopy is unique in providing information at atomic resolution of membrane polypeptides in a native bilayer environment. The technique has been used for investigations of their structure, topology, dynamics and heterogeneous nature in phospholipid bilayers^1^,^2^,^3^,^4^,^5^,^6^,^7^,^8^,^9^. However, NMR suffers from its inherently low signal intensity and the investigation of membrane-associated polypeptides turns out particularly difficult due to the dilution of peptide in lipid (typically around 1-2 mole%) and the increased line width due to inhomogeneous line broadening. Although these limitations are most pronounced when static samples are investigated the large anisotropy of NMR chemical shift, dipolar and quadrupolar interactions has been successively used to determine the structure and/or topology of a number of peptides in oriented lipid bilayers^1^,^2^,^8^,^9^. These could be refined by additional distance restraints for example from magic angle sample spinning (MAS) solid-state NMR spectra^1^,^2^,^8^,^9^.

During the last years considerable progress has been made in dynamic nuclear polarization (DNP) solid-state NMR and the technique has been shown to overcome many of the sensitivity limitations of solid-state NMR spectroscopy^10^,^11^,^12^,^13^. During DNP/solid-state NMR experiments the radicals dispersed in the sample result in a high electron polarization which is transferred to the ^1^H bath through microwave irradiation. In a next step cross-polarization to the heteronuclei assures enhancements of the NMR signal typically by about two orders of magnitude^10^,^14^. Also in the case of membrane samples^4^,^11^,^15^,^16^ or of membrane proteins in cellular environments considerable improvements have been obtained^17^.

In order to optimize the magnetization transfer from the unpaired electrons to the nuclei biradicals of suitable geometries have been designed and incorporated into the sample preparation^18^,^19^,^20^,^21^,^22^,^23^. Magnetization transfer through the cross effect^24^,^25^,^26^ requires that the measurements are performed at cryo-temperatures to assure sufficiently long life times of the electron polarization^27^,^28^,^29^,^30^. Using magic angle oriented sample spinning good DNP signal enhancements were obtained for solid-state NMR measurements of oriented membranes when at the same time it was demonstrated that bilayers made of 1-palmitoyl-2-oleoyl-*sn*-glycero-3-phosphocholine (POPC) retain an oriented bilayer morphology even at 100K and in the presence of biradicals^31^.

Following this proof-of-concept study^31^ a static flat-coil solid-state NMR/DNP probe head has been designed and constructed^32^. Using this new equipment here we assessed for the first time peptides of biological relevance under DNP conditions. We explore if membrane domains retain the overall structure and topology also under low temperature and DNP conditions, and ask the question if significant DNP efficiencies can be obtained from such systems when reconstituted in supported lipid bilayers. One test sequence that we investigated is the PGLa antimicrobial peptide which is particular sensitive to environmental conditions^33^. For comparison a transmembrane helical anchor sequence, which was labeled with ^15^N at five consecutive residues was also studied^34^.

Antimicrobial peptides of the magainin class have been investigated extensively by biophysical approaches, including oriented solid-state NMR spectroscopy, with good evidence that many of them selectively kill bacteria by disrupting their plasma membranes^35^,^36^,^37^,^38^. Whereas magainin 2 and related peptides adopt very stable alignments parallel to the membrane surface under all conditions tested so far^39^,^40^, the PGLa helical sequence (GMASKAGAIA GKIAKVALKA L-amide) has been shown to be highly affected by the detailed composition of the phospholipid membrane, its peptide-to-lipid ratio, the hydration level or the presence of other peptides^33^,^41^. Therefore, this sequence provides a particularly sensitive test case for the potential effects of low temperature conditions used in a variety of physical experiments such as x-ray diffraction or DNP/solid-state NMR^4^,^11^,^15^,^16^.

## Results and Discussion

**Figure 1:**
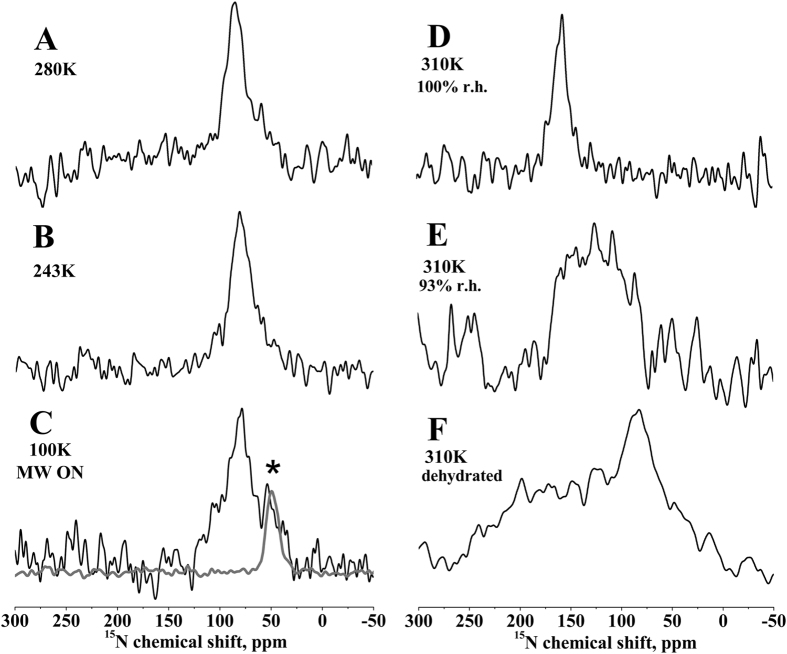
Shows ^15^N solid-state NMR spectra of 2 mole% [^15^N-Ala14]-PGLa in uniaxially oriented DMPC/DMPG 3/1 bilayers (membrane normal parallel to B_o_) as a function of temperature and hydration. (**A**) 280 K, (**B**) 243 K, (**C**) 100 K, (**D**–**F**): 310 K. (**A–C**,**E**) were hydrated at 93% r.h., (**D**) 100% r.h., and (**F**) dehydrated during long measurement (likely around 75% r.h.). The spectrum in panel (**C**) was measured under 7 Watts microwave (MW) irradiation. The gray line in panel (**C**) shows the spectrum of peptide-free POPC vesicles under microwave irradiation. The position of the PC resonance is labeled with an asterisk.

Figure 1 shows the proton-decoupled ^15^N solid-state NMR spectra of 2 mole% [^15^N-Ala14]-PGLa reconstituted into uniaxially oriented 1,2-dimyristoyl-*sn*-3-phosphocholine/1,2-dimyristoyl-*sn*-3-phospho-(1′-*rac*-glycerol) (DMPC/DMPG) 3/1 mole/mole bilayers. At 310K and 93% relative humidity (r.h.) a broad signal intensity with a maximum at 125 ppm is observed (Fig. 1E, line width at half height (LWHH) ca. 80 ppm) in agreement with previous investigations where PGLa has been found to adopt a tilt angle of 53°^33^,^42^,^43^. This tilted configuration has been suggested to be due to the formation of homo-dimers^42^. When the temperature is decreased below the liquid crystalline – gel phase transition temperature of both lipids (T_c_ = 297K and 296K for DMPC and DMPG, respectively) a more homogeneous topology is observed characterized by a ^15^N chemical shift maximum of 87 ppm (Fig. 1A,B; LWHH 23 ppm). This chemical shift coincides with the previously published tilt angle of the PGLa helix of 81° in liquid crystalline DMPC, DMPC/DMPG or POPC bilayers at lipid-to-peptide ratios <1 mole%^42^,^44^. Importantly the good uniform alignment of PGLa persist at lower temperatures (Fig. 1A–C) and even at sub-gel phase temperatures of 100K and in the presence of the biradical PyPol-C16 (Fig. 1C; LWHH 30 ppm)^18^, typical conditions of the commercial DNP/solid-state NMR set-up^45^.

Notably, the solid-state NMR spectrum of this sample was recorded under the effect of 7W microwave irradiation. The sample contains only 0.7 mg of peptide labeled with ^15^N at a single site and did not provide any apparent signal intensity without microwave irradiation even after 2 days of acquisition. Therefore, the enhancement factor could not be determined on this sample, however, spectra from related membranes showed ^15^N signal enhancements of 17-fold under static conditions^32^. When it is taken into account that spinning of the sample around the magic angle (MAS) increases the DNP efficiency several fold^25^,^26^,^45^ this value is among the best enhancement factors measured so far for matrix-free systems, including oriented membranes^30^,^31^,^32^,^46^. This favorable property of PyPol-C16 in the presence of membranes was made possible through the addition of a palmitoyl chain, which assures a better distribution in the oriented lipid bilayer samples devoid of bulk water^18^,^22^,^23^.

Previously ^19^F powder pattern line shapes have been observed for PGLa modified with a CF_3_-carrying Phe-derivative at four different Ala or Ile positions when reconstituted into DMPC/DMPG 3/1 and investigated at 253K or 233K^43^. In mechanically oriented lipid bilayers that are exposed to intense radiofrequency irradiation during solid-state NMR experiments special consideration has to be given to the exact degree of sample hydration over time within the coil volume (the practical aspects are discussed in reference^47^). Therefore, spectra of the same samples shown in Fig. 1A–C,E have also been recorded after equilibration at different degrees of hydration. Whereas Fig. 1D shows a defined intensity at 160 ppm at full hydration^33^, a condition difficult to maintain for oriented lipid bilayers under solid-state NMR conditions^47^, a broad range of ^15^N-H vector (i.e. PGLa helix) alignments are observed upon sample dehydration both at 310 K (Fig. 1F) and at 273 K (not shown). Notably, at the same time the phospholipid membrane remains well aligned with an oriented ^31^P solid-state NMR resonance centered at 35 ppm (not shown).

**Figure 2:**
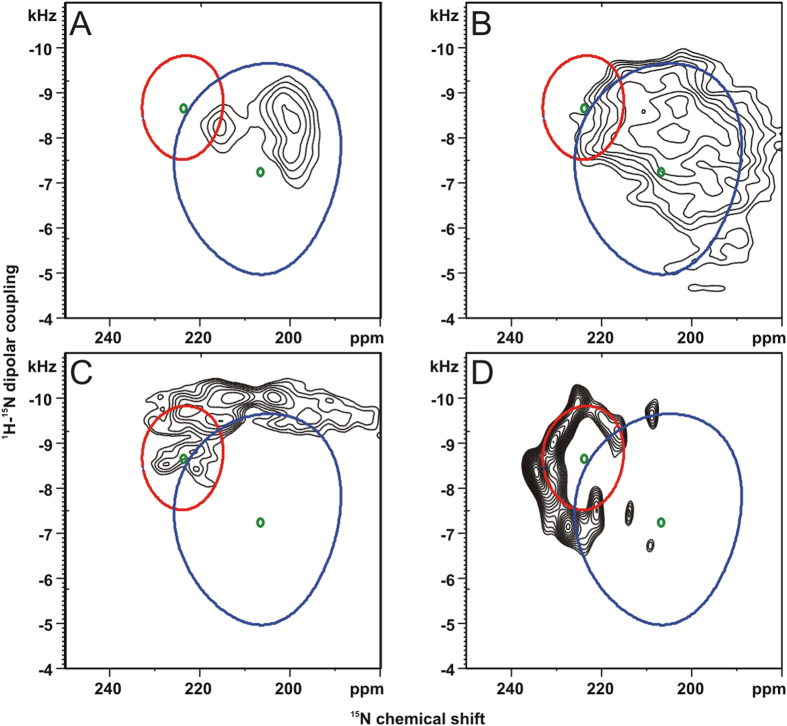
PISEMA spectra of [^15^N_5_]-hΦ19W carrying five consecutive ^15^N labels in uniaxially oriented POPC bilayers. (**A**) ambient temperature (295K), (**B**) 253K and (**C**) 223K. (**D**) nominal 100K (actual sample temperature due to microwave irradiation ~180K). (**A**–**C**) 10 mg of peptide were reconstituted at a peptide-to-lipid ratio (P/L) of 1/35 mole/mole supported by ultra-thin glass plates^47^. (D) DNP/solid-state NMR spectrum of 8.5mg [^15^N_5_]-hΦ19W in the presence of 0.3 mg AMUPol^18^ at P/L = 1/20 oriented onto films of high-density polyethylene^31^,^32^. The simulations show the intensity distributions at static helical tilt angles of 10^o^ (red circle) and 22^o^ (blue circle). Averaging around the helix long axis results in the collapse of the wheel -like pattern into its respective center of gravity (green dots).

In order to investigate the temperature dependence of a transmembrane sequence upon temperature variation and under DNP conditions the [^15^N_5_]-hΦ19W peptide (peptide with a core of 19 hydrophobic residues including a tryptophan; cf. Methods section for details)^34^ was investigated by two-dimensional separated local field spectroscopy where the resolution in the dipolar dimension is enhanced by phase- and frequency switched Lee-Goldberg decoupling of homonuclear ^1^H interactions when at the same time cross polarized with the ^15^N nucleus^48^,^49^. The spectrum obtained at 100 K and under DNP conditions is shown in Fig. 2D where a partial helical wheel that arises from the 5 labeled residues is obvious.

Interestingly at room temperature the same peptide exhibits a much smaller wheel where many intensities coincide, indicating that fast motions around the helix long axis occur (Fig. 2A). Furthermore, the center of mass of the spectra obtained at room temperature and at 100 K is shifted by about 20 ppm. This is probably due to one or several phase transitions of the lipid bilayer concomitant with changes in the hydrophobic thickness of the membranes and a smaller tilt angle at the cryo-temperature. At intermediate temperatures where the lipid is in the gel phase broad intensity distributions are obtained that are difficult to analyze (Fig. 2B,C).

Even though PGLa exhibits a number of membrane topologies the peptide retains an oriented alignment even at sub-gel temperatures and is thereby amenable to solid-state/DNP investigations with good signal enhancements. It should be noted that here we have chosen a bilayer lipid composition where PGLa is particularly sensitive to topological transitions^33^,^42^,^43^. Whereas the gel and sub-gel phases exhibit a more homogenous sample orientation with better resolved lines also at 93% r.h. (Fig. 1A–C) and thereby more suitable conditions for a structural analysis, the topological equilibrium is shifted to a more membrane-inserted configuration in the liquid crystalline bilayer (Fig. 1D,E). Interestingly the low-temperature alignment in DMPC/DMPG closely corresponds to topologies observed under more physiological conditions (membranes made from POPC or from *E. coli* lipid extracts at room temperature)^42^,^44^,^50^. Furthermore, in this work we have characterized hydration as an additional parameter that affects the PGLa topological equilibrium in a sensitive manner. The low temperatures may protect the sample from dehydration when irradiated with strong radio frequency fields and thereby help to maintain a good peptide alignment.

For the transmembrane sequence a 20 ppm shift in the ^15^N chemical shift position indicates a more upright tilt angle (by about 10^o^) which is probably due an increased hydrophobic thickness of the bilayer under such conditions. Whereas the peptide undergoes fast motional averaging and exhibits sharp resonances at room temperature the signals are spread over a wider frequency range under DNP conditions which helps to resolve the NMR intensities from individual sites (Fig. 2A,D). Therefore, these studies show that freezing such motions can lead to inhomogeneous line broadening but can also have beneficial effects in spreading the signals over a broader chemical shift/dipolar coupling range.

Notably, investigating oriented membrane systems at cryo-temperatures by DNP/solid-state NMR is only at its beginnings and there remains a large potential of improvement in changing for example the lipid composition^51^,^52^. For example in the case of PGLa it has been demonstrated that a stable in-plane alignment such as the one observed at low temperatures including DNP conditions (Fig. 1A–C), is obtained in bilayers made from 1-palmitoyl-2-oleoyl- or in *E. coli* phospholipids, which represent much better the natural lipid composition than the dimyristoyl-phospholipids^33^,^50^. Recently, bicellar lipid mixtures have been presented that orient in the magnetic field of the NMR spectrometer even at temperatures of −15 °C^53^, a development which encourages further developments to make this alternative approach for oriented membrane preparations also accessible to DNP solid-state NMR technologies. Furthermore, other sequences exhibit a more stable topology and it can be expected that larger proteins have a more densely packed core^54^,^55^ that is less sensitive to changes in the lipid macroscopic phase properties than the peptides investigated here which further extends the range of applications of oriented DNP/solid-state NMR spectroscopy.

We estimate that in the experiments presented here it takes several minutes before the final cryo-temperature is obtained. However, methods for rapid freezing have been developed also for membrane-samples, thus that the conformational and topological states that rapidly exchange at room temperature are captured and can be investigated by solid-state NMR or electron paramagnetic resonance spectroscopies^56^,^57^. It should therefore be possible to take advantage of the increased sensitivity of DNP solid-state NMR also during investigations of the dynamic features of membrane-associated polypeptides.

## Methods

The phospholipids POPC, DMPC and DMPG are from Avanti Polar Lipids (Alabaster, AL). All commercial material was used without further purification.

### Peptide sequence and label positions

PGLa (GMASKAGAIA GKIAKVALKA L-amide), labeled with ^15^N at the underlined position, and the hydrophobic peptide [^15^N_5_]- hΦ19W (KKKALLALLALAWALALLALLAKKK) were prepared by solid phase peptide synthesis as described previously^34^. At five subsequent positions leucine and alanine labeled with ^15^N were incorporated into the peptide (underlined in the above sequence). The PyPol biradical was prepared as described previously^18^. The preparation of PyPol-C16, a derivative of PyPol bearing a palmitoyl chain, will be described in detail elsewhere.

### Membrane samples for DNP

A homogeneous mixture of lipid, peptide, and radical was obtained by co-dissolving the membrane components in trifluoroethanol. To prepare oriented-phospholipid membranes, the solution was spread onto ultra-thin cover glasses for conventional oriented solid-state NMR measurements (8 × 22 mm, thickness 00; Marienfeld, Lauda-Königshofen, Germany), or for DNP on a High-Density PolyEthylene (HDPE) film (3 × 8 mm, Goodfellow, Cambridge, UK), dried first in air and followed by high vacuum overnight^31^,^47^. Thereafter, the samples were equilibrated during a day in an atmosphere of 93% relative humidity of D_2_O/H_2_O (90/10 by volume). The HDPE film with the membrane layers was carefully folded to fit in the coil and flattened in between two sapphire plates of 3 × 8 mm and 0.5–0.8 mm thickness^32^.

### DNP/solid-state NMR

DNP/solid-state NMR measurements were performed using a Bruker BioSpin wide-bore 9.4 T magnet and an Avance III solid-state NMR spectrometer equipped with a gyrotron producing 263 GHz irradiation, a microwave transmission line, a cooling unit using liquid nitrogen^45^, and a purpose built static solid-state NMR/DNP probe for oriented samples^32^. An adiabatic cross polarization (CP) pulse sequence^58^ was used with a spectral width of 29.8 kHz and acquisition, cross polarization contact and recycle delay times of 8.6 ms, 0.3 ms and 3 s, respectively. The ^1^H π/2 pulse and SPINAL-64 heteronuclear decoupling field strengths B_1_ corresponded to a nutation frequency of 50 kHz^59^. To equilibrate the system before acquisition the sample was exposed to 16 dummy scans. An exponential line-broadening of 100 Hz was applied before Fourier transformation.

The DNP/solid-state NMR Polarization inversion spin exchange at the magic angle (PISEMA) spectrum was recorded on about 9 mg [^15^N_5_]-hΦ19W at a nominal temperature of 100K (where the actual sample temperature depends on the micro wave (MW) irradiation (~180K)). A step-by-step protocol for setting up and analyzing the experiment are given in references^49^,^60^. The effective B_1_ field strength during the SEMA pulse train was 50 kHz. During the spin exchange period the amplitude of the ^1^H B_1_ field was decreased to 40.9 kHz to maintain the Hartmann– Hahn match condition with an effective field along the magic angle of 50 kHz. 128 t_1_ increments were recorded by accumulating 64 scans each (total duration 7h; with no significant improvement after 64 t1 increments/3.5 hours).

Conventional (no DNP) CP and PISEMA experiments were performed using a Bruker Avance solid-state NMR (wide-bore 9.4 T magnet) equipped with efree™ static double resonance bioPE™ probe (Bruker Spectrospin, Karlsruhe, Germany). The temperature was controlled using a BCU-Xtreme unit (Bruker Spectrospin, Karlsruhe, Germany). Proton-decoupled ^15^N solid-state NMR spectra were acquired using an adiabatic CP pulse sequence, a spectral width, acquisition time, CP contact time and recycle delay of 38.5 kHz, 6.7 ms, 0.8 ms and 3 s, respectively. The ^1^H π/2 pulse and SPINAL-64 heteronuclear decoupling field strengths were 35 kHz^59^. Typically 60000 scans were accumulated, and the spectra were zero filled to 4096 points. A 100 Hz exponential line- broadening was applied before Fourier transformation. Spectra were externally referenced with ^15^NH_4_Cl at 40.0 ppm^61^.

The field strength during the SEMA pulse train was 60 kHz, 94 t_1_ increments were recorded by accumulating 2048 scans each (total duration 7 days; with no significant improvement after 64 t_1_ increments/3 days).

## Additional Information

**How to cite this article**: Salnikov, E. S. *et al.* Membrane topologies of the PGLa antimicrobial peptide and a transmembrane anchor sequence by Dynamic Nuclear Polarization / solid-state NMR spectroscopy. *Sci. Rep.* **6**, 20895; doi: 10.1038/srep20895 (2016).
